# A Dairy Herd Case Investigation with Very Low Dietary Cation–Anion Difference in Prepartum Dairy Cows

**DOI:** 10.3389/fnut.2017.00026

**Published:** 2017-06-13

**Authors:** Pedro Melendez, Scott Poock

**Affiliations:** ^1^Department of Veterinary Medicine and Surgery, College of Veterinary Medicine, University of Missouri, Columbia, MO, United States

**Keywords:** DCAD, hypocalcemia, dairy cattle, anionic salts, Urine pH

## Abstract

During the periparturient period, subclinical hypocalcemia (total plasma Ca concentration <2.0 mmol/l) is a potential problem for the dairy cow; consequently, its prevention is essential for success of fertility and productive performance. Dietary cation–anion difference (DCAD) has been defined as the difference in milliequivalents of cations (Na, K) and anions (Cl, S) per kilogram of dry matter (DM) and has a direct impact on blood acid–base metabolism. Diets rich in K and Na induce metabolic alkalosis, interfering with tissue sensitivity to parathyroid hormone, and diets rich in Cl and S (anionic salts) cause metabolic acidosis, reducing the risk of hypocalcemia. Consequently, the use of anionic salts has become a popular method to prevent hypocalcemia in dairy cattle. Monitoring diets with anionic salts can be done by measuring urine pH, with optimal values between 6.2 and 6.8 for Holstein cows. The objective of this report is to present a herd case investigation involving a dairy farm feeding a very low DCAD (−143 mEq/kg DM), expecting improved Ca homeostasis. The diet of −143 mEq/kg (urine pH 5.2–5.8) was changed to a diet with −53 mEq/kg DM (urine pH 6.2–6.8). Blood samples were taken at the time of calving for 10 cows that calved before and then for 10 cows that calved after changing the diet. Cows with extremely low DCAD had Ca concentrations of 2.11 ± 0.22 mmol/l and cows with a more moderated DCAD, 2.11 ± 0.16 mmol/l (*P* > 0.05). Several other blood metabolites (P, Mg, Na, K, Cl, albumin, globulins, blood urea nitrogen, creatinine, and GGT) were also similar between groups. This very low DCAD during the prepartum period may severely compromise animal physiology unnecessarily, with little advantage over normal calcium concentrations at parturition, when compared with a less negative DCAD (−53 mEq/kg DM). Feeding a less negative DCAD ration (−53 mEq/kg DM) did not decrease plasma Ca levels right after parturition compared to a DCAD ration of −143 mEq/kg DM, reinforcing the lack of benefit of a more negative DCAD.

## Introduction

On October 1, 2015, the University of Missouri dairy herd initiated a more systematic cow health monitoring program. The dairy consisted of 200 Holstein lactating cows, milked twice a day, and fed a total mixed ration meeting or exceeding the NRC requirements (1). The ingredients of the ration were corn silage, alfalfa hay, corn grain ground fine, soybean meal, soybean hulls, corn gluten feed, wet brewers, mineral, and vitamin premix. Cows calved in individual maternity pens and moved to a postpartum lot. Every day postpartum cows were monitored for health status until 13 days in milk or beyond if the cow was diagnosed sick. Voluntary waiting period was 60 days. After that cows were subjected to a heat detection protocol and artificially inseminated when they were found in estrus. Pregnancy was diagnosed by ultrasound approximately 30–32 days post breeding. Pregnancy was rechecked at 5 months post breeding and then at dry off (7-month post breeding). Cows were dried off 50 to 70 days before expected parturition. At 3 weeks before expected parturition, dry cows were moved to a prepartum lot where cows were fed anionic salts (Tables 1 and 2) to prevent hypocalcemia and related disorders. Urine pH assessment was, as part of the routine monitoring program, done on a weekly basis. On October 21, 2015, a urine sample from nine cows (30%) of the prepartum cows was obtained according to standard recommendations. The standard recommendation for obtaining a sample is stimulating manually the escutcheon area, collecting a clean, pure, non-contaminated sample. Samples were transported to a laboratory facility cooled on ice within 30 min. Urine pH was assessed by using an electronic pH meter (Benchtop pH/mV Meter-860031, Sper Scientific Direct, Scottsdale, AZ, USA). The results were surprisingly low (<6.0, Table 3) in regard to the recommended range of between 6.2 and 6.8 (2).

**Table 1:** Diets of prepartum cows (kg/cow/day).

Ingredients	Low dietary cation–anion difference (DCAD) diet [−143 mEq/kg dry matter (DM)]		High DCAD diet (−53 mEq/kg DM)	
	As fed	DM	As fed	DM
Brome hay	5.81	5.00	2.50	2.23
Corn silage	8.34	2.72	14.50	4.73
Corn grain ground fine	2.58	2.27	0.80	0.70
Dry cow premix high DCAD^a^	1.56	1.43	–	–
Dry cow premix low DCAD^b^	–	–	1.60	1.42
Soybean meal 47.5 solvent	1.30	1.18	1.20	1.06
Soybean hulls ground	0.49	0.45	2.50	2.27
Wet Brewers	–	–	4.00	0.98

Total	20.12	13.07	27.10	13.42

*^a^A mineral premix formulated based on gluten feed, ammonium chloride, and magnesium sulfate*.

*^b^A mineral premix formulated based on corn grain, soybean meal, and HCl*.

**Table 2:** Nutritional composition of prepartum diets as dry matter (DM) basis using the nutritional Cornell Model (CNCPS 6.55).^a^

Ingredients	Low dietary cation–anion difference (DCAD) diet (−143 mEq/kg DM)	High DCAD diet (−53 mEq/kg DM)
DM (%)	64.90	49.50
Crude protein (%)	14.01	14.51
Soluble protein (%)	3.18	3.51
RUP (%)	5.56	6.51
aNDFom (%)	44.79	44.11
peNDF (%)	36.77	32.20
Starch (%)	19.10	17.42
NFC (%)	28.16	26.50
Fat (%)	2.79	2.89
Ca (%)	1.31	0.94
P (%)	0.30	0.34
Mg (%)	0.41	0.47
K (%)	1.10	1.13
Na (%)	0.07	0.09
S (%)	0.28	0.26
Cl (%)	1.00	0.78
Cu (ppm)	23.00	14.00
Se (ppm)	0.28	0.26
Zn (ppm)	53.00	51.00
Co (ppm)	0.76	0.70
DCAD^b^ (mEq/kg)	−143	−53

*^a^Cornell Net Carbohydrate and Protein System 6.55, Ithaca, New York, NY, USA*.

*^b^DCAD formula (Na + K) – (Cl + S)*.

*RUP, Rumen undegradable protein; aNDFom, ash-free neutral detergent fiber organic matter; peNDF, physically effective NDF; NFC, non-fiber carbohydrates*.

**Table 3:** Mean and range of urine pH for prepartum dairy cows based on high and low negative dietary cation–anion difference (DCAD).

Date	Mean	Range
**Period with low negative DCAD [−143 mEq/kg dry matter (DM)]**		
October 21, 2015	5.48	5.32–5.71
November 11, 2015	5.53	5.11–5.89
December 16, 2015	5.81	5.41–6.12
January 20, 2016	5.72	5.53–6.20
February 24, 2016	5.70	5.53–5.91
**Period with high negative DCAD (−53 mEq/kg DM)**		
March 23, 2016	6.67	5.70–8.23
March 30, 2016	6.90	6.11–8.23
April 6, 2016	6.58	5.88–7.53

*At each date, prepartum group was composed of cows from 1 to 30 days before expected parturition. Every week cows that gave birth left the group and cows that reached 30 days before expected parturition entered the group. At each date, a 20% of the group was sampled for urine pH determination based upon recommendations (2)*.

After this urine pH evaluation, the ration showed a theoretical dietary cation–anion difference (DCAD) of −143 mEq/kg DM (Low DCAD diet), which was consistent with the urine pH obtained. In addition, each cow at parturition received a subcutaneous injection of 500 ml of a commercial product based on Ca borogluconate 23% solution (AgriLabs, St. Joseph, MO, USA). There has not been a case of clinical hypocalcemia in this dairy during the last 11 months. The last clinical case was diagnosed and treated on November 2014. In addition, the incidence of other periparturient disorders was within reported targets (3) and recommended for Holstein cows. This extreme negative DCAD approach, particularly if every cow received a subcutaneous dose of Ca, and the incidence of clinical hypocalcemia was extremely low, was unnecessary and potentially dangerous for the cows (2).

In an effort to determine whether this more negative DCAD formulation had a benefit for the prepartum cows, the dairy decided to attempt a different management approach. A new diet was formulated by one of the authors, targeting a DCAD of −53 mEq/kg DM (high DCAD diet) (Tables 1 and 2). Considering a potential difference of 0.15 ± 0.11 mmol/l of total plasma calcium between cows receiving the more negative DCAD diet and cows receiving the less negative DCAD diet, with 95% confidence and a power of the test of 80%, a sample size of 10 cows per group was calculated (4). Consequently, before changing feed strategy, a blood sample from 10 cows (mean parity number 2.3, range 2–5), within 6 h after parturition and before receiving the subcutaneous Ca product, was obtained to assess blood total Ca and other metabolites (P, Mg, Na, K, Cl, albumin, globulins, BUN, creatinine, and GGT). Chemistry analysis was performed at the veterinary clinical pathology lab of the University of Missouri using an auto analyzer (AU480 Chemistry System, Beckman Coulter, Inc., Brea CA, USA). The metabolic profile of this group of cows was compared with a subsequent group of 10 cows consuming the new prepartum diet (mean parity number 2.4, range 2–6).

Before beginning the new dietary approach, urine pH was tested four more times. The results are shown in Table 3. Based on these results, urine pH consistently mirrored the very low DCAD diet which the prepartum cows have been consuming over time.

On March 15, 2016, the prepartum diet was changed to the new formulation (Tables 1 and 2). An adaptation period of 1 week was considered appropriate since urine pH varies after 72 h of consuming a negative DCAD diet (2). Accordingly, on March 23, 2016, a urine sample from 6 (20%) prepartum cows was taken to evaluate the urine pH. A second urine sample was obtained on March 30, 2016, from 5 (18%) prepartum cows. A third round was obtained on April 06, 2016, from 7 (25%) prepartum cows. Results are reported in Table 3.

Between March 23 and April 10, 2016, a blood sample was obtained from 10 cows within 6 h of parturition before receiving the application of subcutaneous Ca to assess the same plasma metabolites as described previously. Results of plasma metabolites are reported in Table 4. A *t*-test statistical analysis was conducted to determine if the concentration of metabolites before and after changing the DCAD approach was different. In general, the group average for plasma calcium was within reference intervals (2, 5) between groups before and after changing the prepartum nutritional approach. The proportion of cows with subclinical hypocalcemia, defined as <2.0 mmol/l of total calcium (2, 5) was 2/10 cows (20%) for the group fed the more negative DCAD diet and 2/10 cows (20%) for the group fed the less negative DCAD diet (*P* > 0.05). From that time until this case report was completed, no cases of clinical hypocalcemia were diagnosed.

**Table 4:** Mean ± SEM of laboratory results from prepartum dairy cows before and after changing their anionic diet and reference intervals for transition dairy cows.

Metabolite	Low dietary cation–anion difference (DCAD) (−143 mEq/kg) (*n* = 10)	High DCAD (−53 mEq/kg) (*n* = 10)	*P*-value	Reference intervals (19, 20)
Calcium (mmol/l)	2.11 ± 0.22	2.11 ± 0.16	0.50	2.00–2.75
Phosphorus (mmol/l)	1.45 ± 0.42	1.76 ± 0.52	0.16	1.38–2.58
Magnesium (mmol/l)	1.02 ± 0.09	1.08 ± 0.18	0.35	0.83–1.46
Sodium (mEq/l)	143.3 ± 2.66	143.9 ± 5.14	0.74	137.0–148.0
Potassium (mEq/l)	5.38 ± 0.91	6.32 ± 1.74	0.14	3.8–5.8
Chloride (mEq/l)	106.0 ± 2.26	106.1 ± 4.45	0.95	97.0–111.0
Albumin (g/dl)	3.34 ± 0.17	3.49 ± 0.23	0.93	3.0–3.6
Globulins (g/dl)	2.98 ± 0.36	3.22 ± 0.74	0.35	3.0–3.9
BUN (mmol/l)	4.64 ± 1.29	5.39 ± 1.42	0.13	7.14–10.71
Creatinine (μmol/l)	95.29 ± 10.60	94.67 ± 13.26	0.92	88.40–176.8
GGT (IU/l)	12.60 ± 2.27	14.20 ± 5.89	0.21	13.0–33.0

## Background

Calcium is a major mineral that plays a central role in muscle contraction, blood coagulation, enzyme activity, neural excitability, hormone secretion, cell adhesion, and an essential structural component of the skeleton. To maintain a constant concentration of Ca, endocrine control mechanisms have evolved, which primarily consist of the interaction of three major hormones: parathyroid hormone (PTH), calcitonin, and vitamin D (6, 7).

During the periparturient period, hypocalcemia is a potential problem in the dairy cow, as a result of the sudden drain of Ca to colostrum at the onset of lactation. A cow affected with clinical hypocalcemia (<1.25 mmol/l) may present with nervousness and staggering, but usually becomes recumbent and is unable to rise. If blood Ca concentration is not restored quickly, death may occur. 10–50% of cows may develop subclinical hypocalcemia (1.25–2.0 mmol/l) up to 10 days postpartum, affecting organs that have smooth muscle function, such as the uterus, rumen, and the abomasum (2, 5). Consequently, hypocalcemia is a significant risk factor for dystocia, retained fetal membranes, metritis, uterine prolapse, displacement of the abomasum, clinical ketosis, and fatty liver (6). These disorders have been associated with subsequent infertility (2, 6).

Eventually, Ca homeostasis is mediated primarily by PTH, which stimulates bone Ca resorption and renal Ca reabsorption. However, high DCAD or diets rich in K and Na, using the equation (Na + K) – (Cl + S), interferes with tissue sensitivity to PTH (2, 8). As a result, the supplementation of anionic salts during the prepartum period has been used to induce a mild metabolic acidosis and reduce the risk of hypocalcemia (2). These changes are accompanied by a reduction in urinary pH (5, 9). Typical diets fed to cows have a DCAD of about +50 to +250 mEq/kg DM (10, 11). In common feedstuffs, K is the most variable of the ions in the DCAD equation, and it is usually the most important determinant of DCAD in prepartum dairy cows (2, 5, 12).

The strong negative relationship (*r*^2^ = 0.95) between urinary pH and net acid excretion by cows fed the diets containing anionic salts makes the urinary pH evaluation a useful tool to assess the degree of metabolic acidosis imposed by dietary anionic salts (11–13). An advantage of this approach is that it accounts for inaccuracies in mineral analyses, unexpected changes in forage mineral content, and late laboratory results. Urinary pH can be evaluated from a sample representing about 20% of prepartum cows (2). Urine pH values below 5.8 indicate over acidification and DCAD should be increased (2). The optimal urinary pH is between 6.2 and 6.8 for Holstein cows (2). Over 6.8 is considered inadequate acidification and suggests that a lower DCAD is required. Most accurate results will be obtained by collecting urine samples at a standard time, preferably within a few hours after feeding (2, 5, 14).

## Discussion

The intent of this case report was to demonstrate that by changing a nutritional strategy to a less aggressive metabolic acidosis approach, the Ca homeostasis would maintain acceptable levels without compromising the normal physiology of prepartum dairy cows. The use of anionic diets has become a very popular approach to prevent clinical and subclinical hypocalcemia. Due to the use of these strategies, the prepartum cows should develop a mild metabolic acidosis. As a result, the activity of PTH will be enhanced, with an expected response of less hypocalcemia around parturition (2, 15).

There are few studies demonstrating that feeding a negative DCAD for more than 40 days would negatively affect the performance of the periparturient dairy cow and her offspring (16, 17). At this time, the research evidence proposes to feed a DCAD between 0 and −100 mEq/kg DM and reach a urine pH between 6.2 and 6.8 (2). The impact on the incidence of clinical and subclinical hypocalcemia by decreasing the DCAD from +250 to 0 mEq/kg DM and urine pH from >8.0 to 7.0 is dramatic (14). Consequently, what is gained by going to a lower DCAD (< −100 mEq/kg DM) and urine pH (<6.0) is minimal compared to the potential disturbances and damage the cow and fetus can develop (uncompensated metabolic acidosis) (2).

Unfortunately, in this case report, the original nutritional consultant recommended a prepartum diet with a DCAD of −143 mEq/kg DM and urine pH well below 6.0 (between 5.0 and 5.5; Table 3). In addition, based on the new consultant’s experience and communications with other nutritional consultants, there was enough evidence that the approach of prepartum diets with a DCAD below −100 (close to −140 mEq/kg DM) is more common than was thought. Nevertheless, no research evidence has been found demonstrating that by lowering urine pH below 6.0 and the DCAD below −100 mEq/kg DM leads to an extra benefit for the lactating dairy cow (10, 15, 18). The best research evidence supporting this claim is the meta-analysis carried out by Charbonneau et al. (14). In this study, the reduction of DCAD from +300 to 0 mEq/kg DM and urine pH from 8.1 to 7.0 reduced significantly the incidence of clinical hypocalcemia from 16.4 to 3.2%, and reduced DM intake by 11%. Reducing the DCAD below −100 mEq/kg DM and urine pH below 6.0 did not improve significantly the Ca homeostasis and the incidence of hypocalcemia. Furthermore, a more recent study comparing prepartum diets with a DCAD +165 mEq/kg DM versus a DCAD −138 mEq/kg DM reported no differences in Ca status and the incidence of hypocalcemia (15). Consequently, the aforementioned study’s finding suggests that a more moderate DCAD (0 to −50 mEq/kg DM) should not have a negative impact on Ca homeostasis. A study conducted in Minnesota (16) concluded that by feeding a prepartum diet with a DCAD −160 mEq/kg DM either for 42 or 21 days improved substantially the calcium homeostasis when compared to a control group fed a DCAD +120 mEq/kg DM. It should be noted that the prior mentioned study did not compare the more negative DCAD to a more moderate DCAD group (−50 to −100 mEq/kg), leading to wrong conclusions.

The first challenge in the present herd case investigation was to change the manager’s mentality. After all, the manager considered that with the extremely low DCAD strategy, the incidence of clinical hypocalcemia was close to 0% for 11 months. However, the clinical hypothesis of this report was that by changing the strategy, the herd would maintain an adequate Ca status, an extremely low incidence of clinical hypocalcemia. This assumption was based on three major reasons: (i) the dairy was milking twice a day with intermediate milk production (32 kg/cow/day), consequently, the drainage of Ca through the mammary gland is lower than cows milked three times a day (2), (ii) every cow after parturition received a subcutaneous injection of 500 ml of a Ca product supplying extra 10.8 g of Ca to the cow, and (iii) several herds feeding a prepartum diet with a DCAD not lower than −100 mEq/kg DM and urine pH below 6.0, also have extremely low incidence of clinical hypocalcemia and postpartum disorders (11, 14). In the end, the manager of the farm was convinced that this change would bring benefits to the entire herd and a more healthy physiological effect for the cows. As the Table 4 shows, the clinical hypothesis of this report was valid. After changing the nutritional strategy for the prepartum dairy cows (Tables 1 and 2), all the metabolites were statistically the same between the two nutritional strategies and within normal ranges (19, 20). After these results, the manager was entirely satisfied, because the incidence of clinical hypocalcemia and other postpartum disorders were maintained extremely low and within normal ranges reported by this farm during the last 2 years. These findings suggested that the diet with a more negative DCAD was not required to reduce the risk of milk fever, putting the cows at higher risk of an uncompensated metabolic acidosis.

Supporting the data presented in this current case report, a study conducted in Canada (21) demonstrated that a prepartum diet with a DCAD of +12 mEq/kg DM decreased the urine pH from 8.3 to 7.5 when compared with a higher DCAD diet (+145 mEq/kg DM). In addition, this moderated slightly positive DCAD diet also improved the Ca homeostasis and induced a mild but compensated metabolic acidosis. In another study, a prepartum diet with a DCAD of −90 mEq/kg DM also showed that the urine pH decreased to around 7.0 and markedly increased plasma Ca concentration on the day after parturition when compared with a more alkalogenic diet (DCAD +110 mEq/kg DM) (10). However, in a meta-analysis study, it was elucidated that beside a positive impact of a negative DCAD diet on the incidence of hypocalcemia, the levels of Mg and P in the prepartum diet are also important predictors for the risk of hypocalcemia at parturition (22). In the present case study, the plasma concentration of Mg and P were within the reference intervals values reported by the scientific literature (19, 20) and according to the recommended nutritional levels for Mg and P in both prepartum diets (1) (Table 2).

In another study, it was also demonstrated that cows fed either a diet with a DCAD of −120 mEq/kg DM or a diet with a DCAD of −98 mEq/kg DM had similar concentrations of plasma Ca at parturition and higher than a control group (DCAD +220 mEq/kg DM). In addition, no clinical cases of hypocalcemia were reported (23). A prepartum diet with a DCAD of −200 mEq/kg DM and fed for 21, 28, or 41 days before expected parturition showed a plasma Ca concentration around 1.95 ± 0.10 mmol/l, a much lower concentration of Ca than observed in the present case report. Indeed, this concentration falls into the classification of subclinical hypocalcemia (<2.0 mmol/l) and perhaps is due to the extremely low negative DCAD used in that particular experiment (18). Finally, in a study comparing a very low prepartum DCAD diet (−150 mEq/kg DM) with a positive DCAD diet (+100 mEq/kg DM), it was demonstrated that the treatment diet reduced the urine pH dramatically close to 6.0, decreased the DM intake during the prepartum period by 1.5 kg/cow/day, and hardly affected the plasma total Ca concentration at parturition (1.97 versus 1.8 mmol/l) and during the entire postpartum period (24). These findings open a point of discussion on how important is actually the concept of negative DCAD during the prepartum period on Ca metabolism and suggests that there are several other factors involved in the homeostasis of Ca, such as the content of dietary Mg during the prepartum period, that must be taken into account to be successful in the entire performance of the periparturient dairy cow (22). In this particular case report, cows had normal concentrations of Ca before and after changing the dietary strategy. Unfortunately, there was no comparison with a control normal group (positive DCAD diet). Perhaps the difference may have been minimal, and therefore, the strategy change to a less negative DCAD diet was unquestionably effective and less harmful for the cows.

## Concluding Remarks

By feeding a less negative DCAD ration (−53 mEq/kg DM) compared to a more negative DCAD ration (−143 mEq/kg DM), the plasma Ca concentrations right after parturition remained the same within reference intervals and were not statistically different before and after changing the prepartum feed management approach. Plasma P, Mg, Na, K, Cl, albumin, globulins, blood urea nitrogen creatinine, and GGT also remained within reference intervals. This report reinforces the lack of benefit of reducing DCAD beyond −100 mEq/kg DM and putting prepartum dairy cows at higher risk of an uncompensated metabolic acidosis.

## Ethics Statement

This case report is a description of the normal routine of handling of a commercial dairy herd.

## Author Contributions

PM obtained urine and blood samples, formulated the new diet, conducted the statistical analysis, and wrote the manuscript. SP obtained urine and blood samples and helped in the writing of the manuscript.

## Conflict of Interest Statement

The authors declare that the research was conducted in the absence of any commercial or financial relationships that could be construed as a potential conflict of interest.
